# Prediabetes and diabetes among patients presenting with acute stroke or acute coronary syndrome at a tertiary regional referral centre in Kenya

**DOI:** 10.4314/ahs.v26i2.9

**Published:** 2026-06

**Authors:** Beverly Owino, Jasmit Shah, Hasina Visram, Eric Njenga, Nancy Kunyiha

**Affiliations:** 1 Department of Internal Medicine, Aga Khan University, Nairobi, Kenya; 2 Brain and Mind Institute, Aga Khan University, Nairobi, Kenya; 3 Endocrinology and Metabolism, West Ottawa Specialty care, Ontario, Canada

**Keywords:** Prediabetes, Diabetes, Macrovascular, Stroke, Acute Coronary Syndrome

## Abstract

**Background:**

Diabetes and pre-diabetes are significant risk factors for acute coronary syndrome (ACS) and acute primary stroke (APS). Dysglycemia in any context is under-diagnosed or identified late in resource-poor countries such as in sub-Saharan Africa (SSA), especially when the patient is not known to have pre-morbid diabetes.

**Materials and methods:**

We conducted a prospective cross-sectional study at the Aga Khan University Hospital, Nairobi. Inclusion criteria: consenting adults ≥18 years admitted with ACS/APS during the period April 2021-February 2022 inclusive. HbA1C was used to determine the glycemic status. Definition of diabetes and pre-diabetes was based on the American Diabetes Association guidelines.

**Results:**

From a total of 211 patients [144 (81.2%) of African race], the median age of the patients was 58 (49-68) years with a male to female ratio of 2.5:1. 47.4% (n = 100) had ACS and 52.6% (n=111) had APS. The prevalence of dysglycemia was 68.2% (95% CI: 61.5%-74.5%) with the prevalence of pre-diabetes being 30.3% (64/211) and type 2 diabetes 37.9% (80/211). Of the patients with dysglycemia, 47.9% (69/144) had a new diagnosis.

**Conclusion:**

This study shows a remarkably high prevalence of dysglycemia in patients with ACS/APS. The new diagnosis of pre-diabetes prevalence was comparable to diabetes in these patients, adding to the evidence that prediabetes portends significant cardiometabolic consequences and effects over and above the development of type 2 diabetes.

## Introduction

With the expected rise in the cases of diabetes in Africa^1^, the complications related to diabetes particularly macrovascular complications such as stroke and acute coronary syndrome are also expected to increase. This is a public health issue because the population should be able to have easy access to diabetes screening and management.

In a country still burdened by infectious diseases, non-communicable diseases are rapidly increasing and overtaking infectious diseases as major contributors to morbidity and mortality^2^. This duality of disease burden places significant strain on health systems, particularly in settings with limited resources. There are competing interests in management, such as the need for antiretroviral therapy and malaria control, many of which remain donor dependent. The cost of management of diabetes and its complications is high and policymakers face challenges in allocating resources across communicable and non-communicable diseases^3^.

It is therefore imperative to elaborate the burden of disease among patients who already have diabetes-related complications, as this reflects the impact diabetes is already having on the population. Understanding this burden provides insight into disease severity and health system impact. Patients with dysglycemia, including prediabetes, have an elevated cardiovascular risk profile, while those with overt diabetes requiring ongoing care and monitoring for cardiovascular complications.

In Africa there is a challenge when it comes to access of routine screening services because of limited availability of laboratory services like HbA1C. There is also limited access to specialized services to diagnose and manage diabetes-related complications. These gaps contribute to delayed diagnosis and management of dysglycemia and its complications^4^.

This study shows the relationship between dysglycemia and major adverse cardiovascular outcomes. It analyzes patients who already have cardiovascular disease and illustrates the burden of dysglycemia in these patients. It adds to the understanding of diabetes and cardiovascular disease in sub-Saharan Africa and provides evidence related to screening and diagnosis prior to the development of advanced complications. The study also demonstrates the utility of HbA1c in identifying dysglycemia in this setting.

## Methods

### Study design

This was a prospective, cross-sectional study conducted between April 2021 and February 2022.

### Study area

The study was conducted at the Aga Khan University Hospital Nairobi (AKUHN), a tertiary referral hospital accredited as a stroke and myocardial infarction center. The hospital receives patients from across Kenya and the wider East and Central Africa region. On average, approximately 500 patients with acute stroke and myocardial infarction are admitted annually. As part of routine care, all admitted patients undergo baseline metabolic evaluation, including HbA1c, lipid profile, renal function tests, and thyroid function tests.

### Study population

The study population included adult patients admitted to AKUHN with a diagnosis of acute stroke or acute coronary syndrome. Eligibility criteria included all patients at least 18 years old admitted with acute stroke or acute coronary syndrome. Exclusion criteria included patients with brain tumor, head injury, non-atherosclerotic causes of myocardial infarction and vascular malformations as cause of hemorrhagic stroke.

Acute stroke was defined as a sudden onset of focal or global neurological deficit of presumed vascular origin, lasting more than 24 hours or resulting in death, and confirmed by neuroimaging. Acute coronary syndrome was defined as a spectrum of clinical conditions consistent with acute myocardial ischemia or infarction, including ST-elevation myocardial infarction, non–ST-elevation myocardial infarction, and unstable angina, diagnosed based on clinical presentation, electrocardiographic changes, and cardiac biomarkers. These definitions were aligned with the American Heart Association criteria.

### Sample size

The minimum sample size was calculated using the single population proportion formula. Assuming a prevalence of diabetes of 27.9% based on findings from a study by Mohanty et al. 5, a 95% confidence level, and a precision of 5%, the minimum required sample size was 192 participants.

### Sampling procedures

A consecutive sampling approach was used. All patients meeting the eligibility criteria during the study period were approached for participation and enrolled after providing informed consent.

### Study outcomes

The primary outcome was the presence of dysglycemia among patients with acute stroke or acute coronary syndrome.

Dysglycemia was defined based on glycated hemoglobin (HbA1c) levels in accordance with the American Diabetes Association criteria. This included prediabetes, defined as HbA1c 5.7–6.4%, and diabetes, defined as HbA1c ≥6.5% or a prior diagnosis of diabetes. Hypoglycemia was not assessed as part of the dysglycemia definition in this study.

### Data collection

Demographic, clinical and laboratory data were extracted from patient medical records using a structured data collection tool.

### Data Analysis

Data were analyzed using R software. Categorical variables were summarized as frequencies and percentages, while continuous variables were described using means and standard deviations or medians and interquartile ranges, as appropriate. Fischer's exact test and Pearson's Chi-squared test were used to compare categorical variables, while the Kruskal–Wallis test was used for continuous variables. Univariate analysis was performed to assess the association between individual variables and dysglycemia. Multivariable logistic regression analysis was used to identify factors independently associated with dysglycemia. A p-value of less than 0.05 was considered statistically significant.

## Ethical considerations

Ethical approval was sought from the Institutional Ethics and Review Committee at the AKUHN (Ref. No. 2020/IERC - 139) prior to conducting the study. Written informed consent was obtained from the participants prior to enrollment into the study.

## Results

A total of 211 patients were recruited, of whom 52.6% (n=111) were admitted with acute stroke and 47.4% (n=100) had acute coronary syndrome.

### Socio-demographic characteristics

Of the total participants 72.0% (n=152) were males and 68.2% (n=144) were of African origin. When stratified by diabetes status, males comprised a higher proportion of those with diabetes (81.2%) compared to those with pre-diabetes (67.2%) and normal glycemic status (65.7%). The median age of participants was 58 years (IQR: 49–68), with significant variation across groups: individuals with pre-diabetes had the highest median age at 64 years (IQR: 53–71), followed by those with diabetes (60 years; IQR: 51–67), and those with normal glycemia (56 years; IQR: 42–64).

### Clinical Characteristics

Among stroke cases, ischemic stroke was the predominant type across all groups, accounting for 86.5% of all strokes, with no significant differences between the normal, pre-diabetes, and diabetes groups. Similarly, there was no statistically significant variation in the distribution of acute coronary syndrome subtypes. History of myocardial infarction was significantly more common among participants with diabetes (32.5%) compared to those with pre-diabetes (10.9%) or normoglycemia (13.4%) (p=0.002). Notably, participants with diabetes were less likely to report no prior cardiovascular events (52.5%) compared to those with pre-diabetes (78.1%) or normal glycemia (70.1%). Table 1 shows the overall demographic and clinical characteristics comparing normal versus pre-diabetic whereas Table 2 shows the overall demographic and clinical characteristics comparing normal versus diabetes.

**Table 1:** Overall demographic and clinical characteristics comparing normal versus pre-diabetic

Diabetes status			
Variable	Normal	Pre-diabetes	P Value
	N = 67	N = 64	
Gender, n (%)			1.000
Male	44 (65.7)	43 (67.2)	
Female	23 (34.3)	21 (32.8)	
Age in years, Median (IQR)	56 (42 – 64)	64 (53 – 71)	0.004
Ethnicity, n (%)			0.059
African	44 (65.7)	47 (73.4)	
Asian	8 (11.9)	12 (18.8)	
Caucasian	15 (22.4)	5 (7.8)	
Weight in Kgs, Median (IQR)	79 (69 – 90)	77 (70 – 86)	0.680
Height in cms, Median (IQR)	170 (165 – 174)	168 (165 – 172)	0.201
Stroke, n (%)			0.746
Ischemic	36 (83.7)	31 (88.6)	
Hemorrhagic	7 (16.3)	4 (11.4)	
Acute Coronary syndrome, n (%)			0.047
Unstable Angina	0 (0.0)	2 (6.1)	
NSTEMI	11 (42.3)	21 (63.6)	
STEMI	15 (57.7)	10 (30.3)	
Previous cardiovascular event: stroke, n (%)	10 (14.9)	8 (12.5)	0.801
Previous cardiovascular event: Myocardial			
Infarction, n (%)	9 (13.4)	7 (10.9)	0.791
Previous cardiovascular event: None, n (%)	47 (70.1)	50 (78.1)	0.325
Hypertension, n (%)			0.213
Yes	36 (53.7)	42 (65.6)	
No	31 (46.3)	22 (34.4)	
Cigarette smoking, n (%)			0.734
Never	55 (82.1)	54 (84.4)	
Current	8 (11.9)	5 (7.8)	
Previous	4 (6.0)	5 (7.8)	
Wall motion Abnormalities, n (%)			0.475
Akinesia	8 (11.9)	4 (6.2)	
Hypokinesia	14 (20.9)	17 (26.6)	
Normal kinesia	45 (67.2)	43 (67.2)	

**Table 2:** Overall demographic and clinical characteristics comparing normal versus diabetic

Diabetes status			
Variable	Normal	Diabetes	P Value
	N = 67	N = 80	
Gender, n (%)			0.038
Male	44 (65.7)	65 (81.2)	
Female	23 (34.3)	15 (18.8)	
Age in years, Median (IQR)	56 (42 – 64)	60 (51 – 67)	0.009
Ethnicity, n (%)			0.001
African	44 (65.7)	53 (66.2)	
Asian	8 (11.9)	23 (28.7)	
Caucasian	15 (22.4)	4 (5.0)	
Weight in Kgs, Median (IQR)	79 (69 – 90)	78 (73 – 85)	0.713
Height in cms, Median (IQR)	170 (165 – 174)	169 (165 – 172)	0.311
Stroke, n (%)			
Ischemic	36 (83.7)	29 (87.9)	0.747
Hemorrhagic	7 (16.3)	4 (12.1)	
Acute Coronary syndrome, n (%)			0.234
Unstable Angina	0 (0.0)	4 (8.3)	
NSTEMI	11 (42.3)	24 (50.0)	
STEMI	15 (57.7)	20 (41.7)	
Previous cardiovascular event: stroke, n (%)	10 (14.9)	12 (15.0)	1.000
Previous cardiovascular event: Myocardial Infarction, n (%)	9 (13.4)	26 (32.5)	0.011
Previous cardiovascular event: None, n (%)	47 (70.1)	42 (52.5)	0.042
Hypertension, n (%)			0.015
Yes	36 (53.7)	59 (73.8)	
No	31 (46.3)	21 (26.2)	
Cigarette smoking, n (%)			0.948
Never	55 (82.1)	64 (80.0)	
Current	8 (11.9)	11 (13.8)	
Previous	4 (6.0)	5 (6.2)	
Wall motion Abnormalities, n (%)			0.029
Akinesia	8 (11.9)	6 (7.5)	
Hypokinesia	14 (20.9)	33 (41.2)	
Normal kinesia	45 (67.2)	41 (51.2)	

### Association with Dysglycemia

Among the 211 participants, 68.2% (n=144) had dysglycemia (pre-diabetes or diabetes), while 31.8% (n=67) were non-diabetic. Participants with dysglycemia were significantly older, with a median age of 61.0 years (IQR: 51.8–70.2) versus 56.0 years (IQR: 42.0–64.0) (p=0.002). Ethnicity was significantly associated with dysglycemia status (p = 0.001): Asians had the highest proportion of dysglycemia (81.4%), followed by Africans (68.5%), while Caucasians were more likely to be non-diabetic (62.5%). A higher proportion of participants with hypertension had dysglycemia (73.7%) compared to those without hypertension (58.1%) (p=0.030). Median triglyceride levels were significantly higher among those with dysglycemia (1.6 mmol/L, IQR: 1.0–2.0) compared to non-diabetics (1.0 mmol/L, IQR: 0.8–1.6) (p=0.001).

Similarly, HDL cholestero was significantly lower in those with dysglycemia (median: 1.0 mmol/L, IQR: 0.8–1.2) than in non-diabetics (1.1 mmol/L, IQR: 0.9–1.5) (p=0.002). Table 3 summarizes the associations between demographic and clinical characteristics with dysglycemia. In multivariable logistic regression analysis, increasing age, Caucasian ethnicity, hypertension, and higher triglyceride levels were independently associated with dysglycemia. Compared with other ethnic groups, Caucasian ethnicity was associated with a substantially lower likelihood of dysglycemia, conferring an 87% reduction in odds (OR 0.13; 95% CI: 0.04–0.39). Higher triglyceride levels were associated with increased odds of dysglycemia, with each unit increase corresponding to an odds ratio of 2.44 (95% CI: 1.44–4.48). Similarly, increasing age was associated with higher odds of dysglycemia, with a 5% increase in odds per year of age (OR 1.05; 95% CI: 1.02–1.08). Although not statistically significant, a prior history of myocardial infarction, higher body mass index category (overweight and obese), and the presence of hypokinesia were associated with increased odds of dysglycemia.

**Table 3:** Associations with Dysglycemia among demographic and clinical characteristics

Presence of Diabetes				
		No Dysglycemia	Dysglycemia	P-value
Variable	Categories	n (%)	n (%)
	Total	67 (31.8)	144 (68.2)	
Gender	Male	44 (28.9)	108 (71.1)	0.188
	Female	23(39.0)	36 (61.0)	
Age	Median (IQR)	56.0 (42.0-64.0)	61.0 (51.8-70.2)	0.002
Ethnicity	African	44 (30.6)	100 (68.5)	0.001
	Asian	8 (18.6)	35 (81.4)	
	Caucasian	15 (62.5)	9 (37.5)	
Weight (Kgs)	Median (IQR)	79.0 (69.2-89.5)	78.0 (70.0-85.4)	0.988
Height (cm)	Median (IQR)	170.0 (165.0-174.0)	168.5 (165.0-172.0)	0.192
Previous stroke	No	57 (31.5)	124 (68.5)	0.835
	Yes	10 (33.3)	20 (66.7)	
Previous MI	No	58 (34.3)	111 (67.7)	0.138
	Yes	9 (38.1)	33 (61.9)	
Previously no condition	No	20 (27.8)	52 (72.2)	0.438
	Yes	47 (33.8)	92 (66.2)	
Hypertension	No	31 (41.9)	43 (58.1)	0.030
	Yes	36 (26.3)	101 (73.7)	
Cigarette smoking	Never	55 (31.8)	118 (68.2)	0.999
	Current	8 (33.3)	16 (71.4)	
	Previous	4 (28.6)	10 (35.7)	
Creatinine	Median (IQR)	88.0 (68.0-106.5)	80.5 (58.5-98.2)	0.072
Total cholesterol	Median (IQR)	4.4 (3.2-5.5)	4.2 (3.4-5.5)	0.920
Total cholesterol	≤ 5.18	47 (32.9)	96 (67.1)	0.639
	>5.18	20 (29.4)	48 (70.6)	
LDL Cholesterol	Median (IQR)	2.9 (2.0-3.7)	2.9 (1.9-3.9)	0.994
LDL Cholesterol	≤ 2.6	28 (30.8)	63 (69.2)	0.881
	>2.6	39 (32.5)	81 (67.5)	
Triglycerides	Median (IQR)	1.0 (0.8-1.6)	1.6 (1.0-2.0)	0.001
Ejection fraction	Median (IQR)	55.0 (45.0-60.0)	55.0 (45.0-60.0)	0.246
HDL Cholesterol	Median (IQR)	1.1 (0.9-1.5)	1.0 (0.8-1.2)	0.002
Wall motion Abnormalities	Akinesia	8 (44.4)	10 (55.6)	0.081
	Hypokinesia	14 (21.9)	50 (78.1)	
	Normal kinesia	45 (34.9)	84 (65.1)	
BMI	Healthy	27 (42.2)	37 (57.8)	0.080
	Over-weight	23 (25.3)	68 (74.7)	
	Obesity	17 (30.4)	39 (69.6)	

## Discussion

We found that seven out of ten patients who presented with stroke and ACS had dysglycemia. Among patients with a history of cardiovascular disease, the majority were found to have dysglycemia. Regarding gender distribution, the male-to-female ratio was 2.5:1, which aligns with findings from various studies evaluating the impact of gender on cardiovascular disease (CVD). Several factors, including the protective role of hormones, have been proposed to explain this disparity^6^.

Patients with dysglycemia exhibited lower HDL and higher triglyceride levels compared to those with normal glucose levels. This study also demonstrated that hypertension was more prevalent among patients with dysglycemia than in normoglycemic individuals. Additionally, a greater proportion of overweight and obese patients were found to have dysglycemia. These findings are consistent with the Prospective Studies Collaboration, which showed that increased BMI is associated with elevated CVD risk^7^.

Dysglycemia was also associated with a higher prevalence of wall motion abnormalities (hypokinesia and akinesia) on 2D transthoracic echocardiography; however, no significant difference was observed in ejection fraction between the dysglycemic and normoglycemic groups.

These data suggest that macrovascular complications may begin early in the dysglycemia continuum. The prevalence of both diabetes and prediabetes was notable among patients with major adverse cardiovascular events, a finding echoed in the glucose tolerance in patients with acute myocardial infarction, Euro Heart Survey, and China Heart Survey^8^. Prior research has shown that prediabetes shares similar features with diabetes, including hypertriglyceridemia, reduced HDL, and accelerated atherogenesis^9^. Prediabetes has also been shown to double the risk of recurrent stroke, a finding that our study confirms, further emphasizing that prediabetes should be managed aggressively with both lifestyle and pharmacological interventions^10^.

Diabetes, a well-established risk factor for stroke and ACS, has been referred to as a coronary heart disease equivalent. Our findings contribute to the existing body of knowledge by examining its impact on cardiovascular disease within an African population. Diabetes is also known to significantly increase the risk of recurrent cardiovascular events^11^. In our study, among patients with stroke and ACS, the majority had overt diabetes, and most patients who experienced recurrent events were also diabetic. Forty-three percent of diabetic patients had hypertension, a proportion lower than in other studies, which have reported rates as high as 70%. Although both conditions independently raise CVD risk, their coexistence significantly amplifies this risk^12^. Hypokinesia was a common echocardiographic finding among diabetic patients. This could be attributed to the longer latent period of type 2 diabetes prior to diagnosis, or the possibility that these individuals had experienced silent ischemia before presenting with cardiovascular complications^13^.

## Conclusion

This study demonstrates a high prevalence of dysglycemia among patients admitted with acute stroke and acute coronary syndrome, highlighting a substantial burden of abnormal glycaemic states in individuals with established cardiovascular disease. Dysglycemia was commonly accompanied by other cardiometabolic abnormalities, contributing to increased cardiovascular risk. These findings underscore the importance of recognizing dysglycemia as a key comorbidity in patients with acute cardiovascular events and reflect the growing public health burden of diabetes in resource-limited settings.

## Figures and Tables

**Table 4:** Logistic regression parameter estimates of factors associated with dysglycemia summarizes the logistic regression results

Univariable		Multivariable			
Variable	Categories	OR (95%CI)	P-value	OR (95%CI)	P-value
Gender	Male				
	Female	0.64 (0.34-1.20)	0.161	0.66 (0.31-1.45)	0.296
Age	Median (IQR)	1.03 (1.01-1.06)	0.003	1.05 (1.02-1.08)	0.003
Ethnicity	African				
	Asian	1.93 (0.86-4.77)	0.129	0.90 (0.34-2.55)	0.838
	Caucasian	0.26 (0.10-0.64)	0.004	0.13 (0.04-0.39)	< 0.001
Previous MI	No				
	Yes	1.92 (0.89-4.50)	0.112	1.08 (0.41-2.99)	0.884
HTN	No				
	Yes	2.02 (1.11-3.69)	0.021	1.05 (0.48-2.23)	0.906
BMI category	Healthy				
	Overweight	2.16 (1.09-4.31)	0.028	1.76 (0.80-3.90)	0.161
	Obesity	1.67 (0.79-3.61)	0.181	1.85 (0.76-4.63)	0.182
Creatinine	Median (IQR)	0.99 (0.98-1.00)	0.083	1.01 (0.99-1.02)	0.372
HDL Choleterol	Median (IQR)	0.23 (0.09-0.53)	0.001	0.44 (0.16-1.15)	0.099
Triglycerides	Median (IQR)	2.06 (1.35-3.34)	0.002	2.44 (1.44-4.48)	0.002
Wall motion Abnormalities	Akinesia				
	Hypokinesia	2.86 (0.93-8.69)	0.062	1.77 (0.46-6.64)	0.395
	Normal kinesia	1.49 (0.54-4.05)	0.431	0.89 (0.25-3.02)	0.853
